# MicroRNA‐576‐5p enhances the invasion ability of trophoblast cells in preeclampsia by targeting TFAP2A

**DOI:** 10.1002/mgg3.1025

**Published:** 2019-11-08

**Authors:** Xiaoning Wang, Shiyuan Peng, Kun Cui, Fangjuan Hou, Jie Ding, Ali li, Mingxia Wang, Li Geng

**Affiliations:** ^1^ Department of Medical The 987 Hospital of the PLA Joint Logistics Support Force Baoji Shannxi China

**Keywords:** invasion, miR‐576‐5p, preeclampsia, TFAP2A

## Abstract

**Background:**

Preeclampsia (PE) is a common pregnancy‐related syndrome characterized by hypertension and proteinuria, and a major cause of maternal mortality. Therefore, there is an urgent need to identify early biomarkers of PE. The aim of the present study was to identify the functions of miR‐576‐5p in PE.

**Methods:**

Effects of miR‐576‐5p and transcription factor AP‐2α (*TFAP2A*) on invasion of human trophoblast HTR8/SVneo cells were investigated. Real‐time quantitative polymerase chain reaction (RT‐qPCR) and western blotting were used to assess the expression of miR‐576‐5p, *TFAP2A*, E‐cad, and Vimentin in PE tissues and cells. Additionally, immunofluorescence was used to detect the expression of TFAP2A in PE trophoblastic tissue. Subsequently, constructed miR‐576‐5p mimics, miR‐576‐5p inhibitor, and siRNA‐TFAP2A plasmids were transfected into HTR8/SVneo cells for further experiments, including a CCK‐8 assay for cell proliferation, Transwell assay for cell invasion and the luciferase reporter gene system was employed for target verification.

**Results:**

A lower expression of miR‐576‐5p and a higher expression of TFAP2A were identified in PE rats. E‐cadherin was highly expressed while Vimentin was downregulated. Further statistical analysis indicated that cell proliferation of HTR8/SVneo cells decreased in the miR‐576‐5p inhibitor group and increased in the miR‐576‐5p mimics and siRNA‐TFAP2A groups. miR‐576‐5p inhibitor suppressed cell invasion, and miR‐576‐5p mimics and siRNA‐TFAP2A improved cell invasion. The analysis of luciferase reporter demonstrated a decreased luciferase activity in miR‐576‐5p mimics group compared with control group, which indicates that TFAP2A may be a target of miR‐576‐5p. Interference of TFAP2A could downregulate E‐cadherin and upregulate Vimentin expression.

**Conclusion:**

Overexpression of miR‐576‐5p and knockdown of TFAP2A may elevate cell proliferation and invasion of human trophoblast cells in vitro. Therefore, miR‐576‐5p may be used as a notable biomarker for the diagnosis, prevention, and treatment of PE. miR‐576‐5p targeting TFAP2A deserve further investigation in order to explore their potential role in PE.

## Graphical abstract

Preeclampsia (PE) is a common pregnancy‐related disease characterized by hypertension and proteinuria, and a major cause of maternal mortality. Therefore, there is an urgent need to identify early biomarkers of PE. The aim of the present study was to identify the functions of miR‐576‐5p in PE.

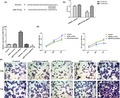

## 1 INTRODUCTION

Preeclampsia (PE) is a common health problem with adverse effects for fetus and mother (Magee et al., 2008), which is a systemic syndrome that results from placental defects, with hypertension, proteinuria, and edema as its major characteristics (Karumanchi, Maynard, Stillman, Epstein, & Sukhatme, 2005). During normal pregnancy, specialized placental cells, known as trophoblasts, acquire tumor‐like properties and invade deep into the uterine decidua inner myometrium (Fisher, 2004; Noris, Perico, & Remuzzi, 2005). However, trophoblasts fail to invade the uterus properly in the PE placentas (Cui et al., 2012). Investigations on the molecular mechanisms related to insufficient trophoblastic invasion will help to understand the pathogenesis of PE.

The previous studies had suggested that serum‐based microRNAs (miRNAs) might be potential biomarkers or effective therapy for early detection, diagnosis, and follow‐up of severe PE (Lycoudi, Mavreli, Mavrou, Papantoniou, & Kolialexi, 2015; Ura et al., 2014). Target messenger RNAs (mRNAs) were subsequently degraded by the formation of RNA‐induced silencing complex, which suggested that miRNAs might control a range of different biological functions including cellular differentiation, proliferation, and viability (Van Wynsberghe, Chan, Slack, & Pasquinelli, 2011). The miR‐576‐5p was proved to contribute to the regulation of postnatal growth and induced cardiometabolic diseases related to a mismatch between pre‐ and postnatal weight gain (Mas‐Pares et al., 2019). Previous researcher showed that miR‐576‐5p enhanced melanoma cell invasion in vitro (Kordass et al., 2018). Additionally, TFAP2A family members were proved to function to modulate expression of lipid droplet proteins and exert pro‐proliferative effects in developmental and in pathological contexts by inducing the accumulation of lipid droplets, which was related with the regulation of neutral lipid metabolism (Scott, Vossio, Rougemont, & Gruenberg, 2018). Lipid metabolism also played a role in the pathogenesis of PE. The present study aimed to examine whether TFAP2A that underwent alterations in expression during the course of PE was regulated by miR‐576‐5p.

The human villus extracellular trophoblast (EVT) cells invaded into the deep part of uterine decidua and gradually replaced the endothelial cells of uterine spiral artery to maintain the stability of maternal blood perfusion (McKinnon, Chakraborty, Gleeson, Chidiac, & Lala, 2001). As the first embryonic cells were in contact with maternal blood, EVT had important physiological significance in trophoblast cell migration and adhesion, embryo implantation, maternal‐infant material exchange, embryo immune tolerance, etc. (Bechi et al., 2013; Graham et al., 1993). For it was very similar to EVT in nature and phenotype, HTR8/SVneo cells were often used as an in vitro model of EVT cells to carry out various related studies, such as the influencing factors of cell invasiveness and migration as well as the relationship with pregnancy‐related diseases.

In the present study, HTR8/SVneo cells were selected as the in vitro model of human EVT cells. Then, we measured the expression of miR‐576‐5p in chorionic trophoblastic tissue and HTR8/SVneo cells and explored whether miR‐576‐5p might affect cell viability and invasion of human chorionic trophoblast cells by regulating TFAP2A. The results indicated that miR‐576‐5p might affect cell proliferation and cell invasion in HTR8/SVneo cells in vitro. The results of the present study may be, therefore, the basis for further studies to examine the pathophysiological mechanism and identify PE biomarkers in order to improve the diagnosis, prevention, and treatment of PE.

## 2 MATERIALS AND METHODS

### 2.1 Ethical compliance

The study and all associated procedures were approved by the Local Ethics Committee of the 987 hospital of the PLA joint logistics support force.

### 2.2 Animals

Sprague Dawley rats weighing 180–230 g were used in the present experiment. All rats were housed in specific pathogen‐free conditions with free access to food and water. The female rats were caged for 24 hr with the male rat and mating was confirmed by the presence of a vaginal plug and spermatozoa in the vaginal smear. The day on which insemination was detected was designated as the day 1 of pregnancy. Pregnant rats were randomly divided into two groups: model group (PE) and pregnancy control group (Control), with five rats in each group. All efforts were made to minimize animal suffering and to reduce the number of animals used.

### 2.3 Induction of PE symptoms

Beginning on the 12th day of pregnancy, gravid rats were given Nω‐nitro‐L‐arginine methyl ester (L‐NAME) (Sigma‐Aldrich) with subcutaneous injection of 250 mg/(kg·d) for 4 days to produce arterial hypertension and proteinuria. Rats in the control group were subcutaneously injected with the same amount of normal saline under the same conditions. Rats were executed on the 16th day of pregnancy. The chorionic trophoblastic tissue was isolated from the placenta of pregnant rats.

### 2.4 Immunofluorescence staining

The chorionic trophoblastic tissue sections were washed with PBS three times. After blocking in 10% milk for 1 hr at 37°C followed by incubation with TFAP2A primary antibodies (ab108311, 1:250) at 4°C overnight. After washing with PBS, the sections were incubated with a 1:200 dilution of a fluorescent tag (Alexa Fluor 488; Thermo Fisher Scientific) and conjugated with secondary antibodies for 30 min in the dark. Next, the sections were treated with DAPI (2.5 μg/ml) for 20 min, washed with PBST, covered with an antifade mounting medium, and placed onto microscope slides. Finally, the location of TFAP2A was measured using fluorescence microscope equipped with a digital camera (Q Imaging, Burnaby, BC, Canada). Photographs were taken with 200 × magnification.

### 2.5 Cell culture and treatment

The human chorionic trophoblast cells (HTR8/SVneo) were obtained from Shanghai kanglang biotechnology co., LTD (China). The cells originated from human first trimester placenta and immortalized by transfection with a cDNA construct that encodes the simian virus 40 large T antigen (Graham et al., 1993). HTR8/SVneo cells were maintained in RPMI 1,640 solution (Sigma) supplemented with 10% fetal bovine serum (FBS). The medium was supplemented with 100 U/ml penicillin and 100 U/ml streptomycin (Sigma). HTR8/SVneo cells were seeded in 24‐well plates in humidified 5% CO_2_ at 37°C until reaching 70%–80% confluence.

### 2.6 Cell transfection

The TFAP2A siRNA, siRNA control, miR‐576‐5p mimics, miR‐576‐5p inhibitor, and their negative control (NC) were synthesized by Shanghai GenePharma, Ltd. (Shanghai, China). Then transfection was conducted in HTR8/SVneo cells with above vectors using Lipofectamine 2000 (Invitrogen, Carlsbad, CA) according to the manufacturer's protocol. After 48 hr of transfection, cells were collected for subsequent tests. Quantitative real‐time PCR (qRT‐PCR) and western blotting were performed to test the transfection efficiencies, respectively.

### 2.7 Total RNA isolation and real‐time quantitative polymerase chain reaction (RT‐qPCR)

Total RNA in chorionic trophoblastic tissue and HTR8/SVneo cells was isolated using the TRIzol® reagent (Invitrogen; Thermo Fisher Scientific, Inc., Waltham, MA, USA). For TFAP2A, E‐cad, Vimentin, and miR‐576‐5p expression analysis, RNA was used for the synthesis of complementary DNA (cDNA) using TaqMan Reverse Transcription Kit or TaqMan microRNA Reverse Transcription Kit (Thermo Fisher Scientific, Waltham, MA, USA). RT‐qPCR was performed using a SYBR Green RT‐PCR Kit and Thermal Cycler Dice Real Time System (Takara Bio, Shiga, Japan) following the amplification instructions. The thermal cycling conditions were set as follows: Initial denaturation at 95°C for 10 min, followed by 32 cycles of denaturation at 95°C for 15 s, annealing at 60°C for 30 s, and extension at 75°C for 40 s. U6 and GAPDH were used as the internal control for the expressions of miRNA and mRNA. All primer sequences are presented in Table 1. The experiments were performed three times and data were calculated with 2^−ΔΔCt^ method.

**Table 1:** Primers used for quantitative polymerase chain reaction

Gene	Forward primer (5'‑3')	Reverse primer (5'‑3')
TFAP2A	AGGTCAATCTCCCTACACGAG	GGAGTAAGGATCTTGCGACTGG
E‐cad	ATTTTTCCCTCGACACCCGAT	TCCCAGGCGTAGACCAAGA
Vimentin	GCCCTAGACGAACTGGGTC	GGCTGCAACTGCCTAATGAG
GAPDH	ATCATCCCTGCCTCTACTGG	GTCAGGTCCACCACTGACAC
miR−576−5p	TTGGGTCAAGAGTCAGAAGTTT	TGGCTTCTACTTGTCCTTTCC
U6	AAAGCAAATCATCGGACGACC	GTACAACACATTGTTTCCTCGGA

### 2.8 Western blotting

Total proteins in chorionic trophoblastic tissue and HTR8/SVneo cells were extracted using RIPA lysis buffer according to the manufacturer's instruction (Beyotime Biotechnology, Shanghai, China) and quantified using bicinchoninic acid (BCA) protein assay kit (Sigma). Proteins were resolved by 10% sodium dodecyl sulfate polyacrylamide gel electrophoresis (SDS‐PAGE) and then transferred to polyvinylidene difluoride (PVDF) membranes. After blocked with 5% nonfat milk in Tris‐buffer saline containing 0.1% Tween 20 (TBST) for 1 hr at room temperature, the membranes were incubated with primary antibodies against TFAP2A (ab108311, 1:1,000), E‐cad (ab76055, 1:500), Vimentin (ab8978, 1:1,000), or GAPDH (ab8245, 1:5,000) overnight at 4°C and then interacted with horseradish peroxidase (HRP)‐conjugated secondary antibodies (ab205718, 1:10,000 or ab205719, 1:10,000, Abcam) for 2 hr at room temperature. The protein bands were detected by chemiluminescence. Densitometry of the resulting bands was performed using ImageJ software.

### 2.9 Cell viability assay

To assess the function of miR‐576‐5p in HTR8/SVneo cells in vitro, a cell counting kit‐8 (CCK‐8) assay was used to measure cell viability according to the manufacturer's instructions. Cells were inoculated in 96‐well plates at a density of 2 × 10^4^ cells per well for 24 hr. Then, 15‐μl CCK‐8 reagent was added to each well at 24, 48, or 72 hr after transfection. After the incubation at 37°C for 2 hr, the absorbance was measured at 490 nm using a microplate reader (Bio‐Rad, USA). Every sample was prepared in triplicate and the experiment was repeated three times.

### 2.10 Cell invasion assay

For cell invasion assay, HTR8/SVneo cells were resuspended in serum‐free medium and subsequently plated into the upper side of a 24‐well Transwell Matrigel chambers with 20‐µl Matrigel‐coated membranes. The medium containing 10% FBS was added into the lower chambers. Each group was added to the 8‐mm pore size upper transwell chambers (BD Biosciences, San Jose, CA). After 24 hr incubation, the noninvasive cells on the upper sides of the chambers were removed with a cotton swab. Then, 4% paraformaldehyde and 0.1% crystal violet were used to stain the cells on the lower sides of the chambers. After washing with PBS for three times, the photographs were taken by an inverted microscope (Leica, Germany).

### 2.11 Dual‐luciferase assay

The putative binding sites of miR‐576‐5p and TFAP2A were predicted by TargetScan software online. The 3′ untranslated regions (3′UTR) sequences containing wild‐type (WT) or mutant (MUT) binding sites of TFAP2A were subcloned into a PGL3 luciferase reporter vector (Promega, Madison, WI, USA) to generate the TFAP2A‐WT or TFAP2A‐MUT plasmids, respectively. The miR‐576‐5p mimics were cotransfected with TFAP2A‐WT or TFAP2A‐MUT plasmids into HTR8/SVneo cells. The luciferase activities were analyzed using Dual‐Luciferase Assay Kit (Promega) after transfection for 48 hr.

### 2.12 Statistical analysis

Data were expressed as the mean values ± standard deviation (*SD*) from three independent experiments. Data were analyzed by Student's t test or one‐way ANOVA, followed by Tukey‐Kramer multiple comparison test using SPSS 12.0 software (IBM SPSS, Chicago, IL, USA). The statistically significant was regarded as *p* < .05.

## 3 RESULTS

### 3.1 Expression of miR‐576‐5p and TFAP2A in trophoblastic tissue of PE rats

**Figure 1:**
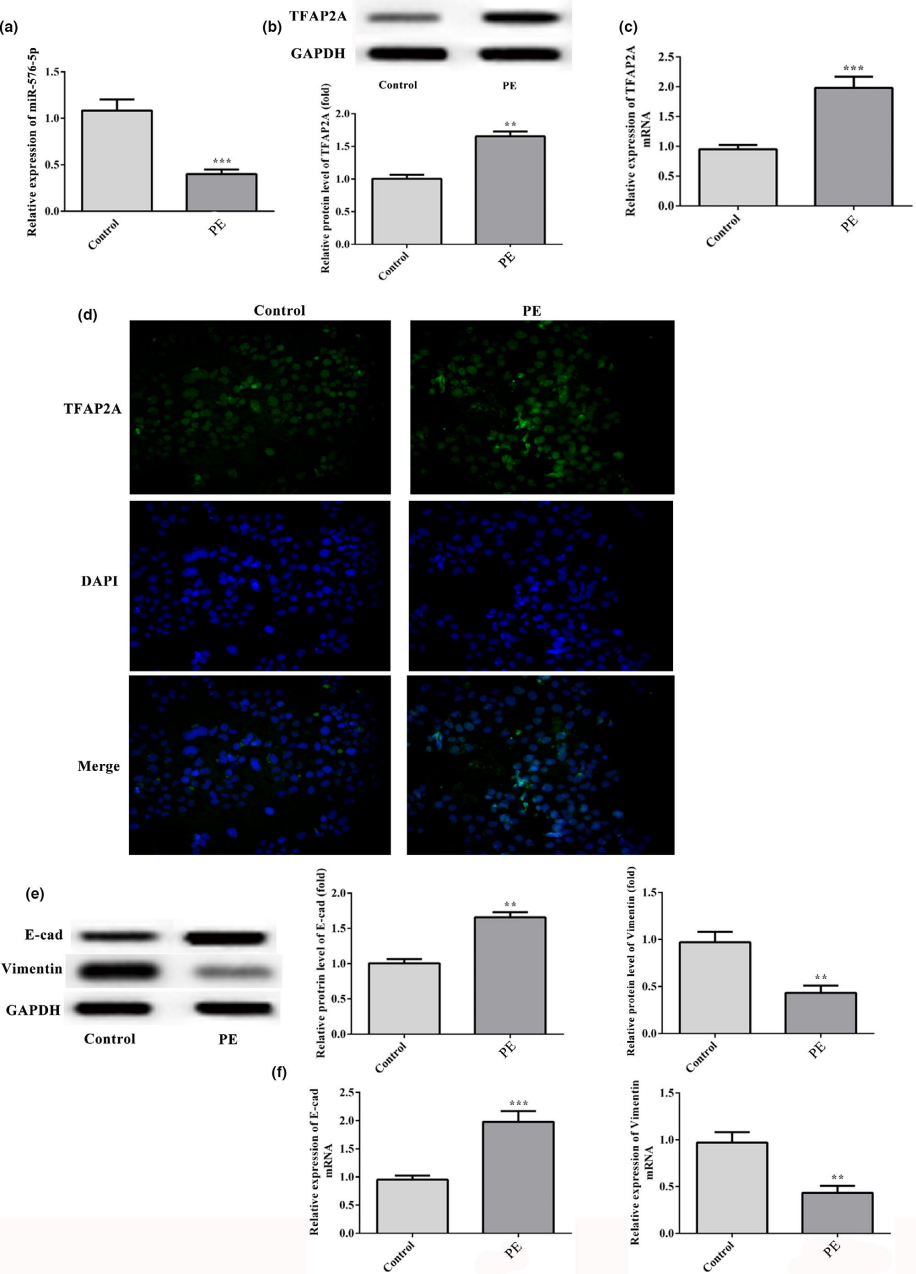
Expression of miR‐576‐5p, TFAP2A, E‐cad, and Vimentin in trophoblastic tissue of PE rats. Expression of (a) miR‐576‐5p, (b) the protein level of TFAP2A, and (c) the mRNA level of TFAP2A were detected using RT‐qPCR and western blot assays. (d) Representative images of immunofluorescence for TFAP2A expression in trophoblastic tissue. The protein level (e) and mRNA level (f) of E‐cad and Vimentin. Data are presented as the mean ± *SD*. All experiments were repeated independently three times. **p < .01 and ***p < .001 versus control group

Expression levels of miR‐576‐5p and TFAP2A in trophoblastic tissue were measured using RT‐qPCR and western blotting. The results showed significantly decreased expression of miR‐576‐5p in PE rats compared with control (*p* < .001; Figure 1a). However, the protein and mRNA levels of TFAP2A showed a sharp increase in PE rats when compared with control group (*p* < .01 and *p* < .001; Figure 1b and c). Similarly, results of immunofluorescent staining presented that TFAP2A expression (green fluorescence) was significantly elevated in trophoblastic tissue of PE rats comparing to control (Figure 1d).

### 3.2 Expression of invasive proteins in trophoblastic tissue of PE rats

As shown in Figure 1e, western blotting was conducted to detected the proteins expression of E‐cad and Vimentin in trophoblastic tissue, and found a dramatic increase of E‐cad and a decrease of Vimentin in PE rats when compared with control group (*p* < .01; Figure 1e). Meanwhile, results from RT‐qPCR were consistent with that in western blotting assay (Figure 1f).

### 3.3 TFAP2A is a target gene of miR‐576‐5p

**Figure 2:**
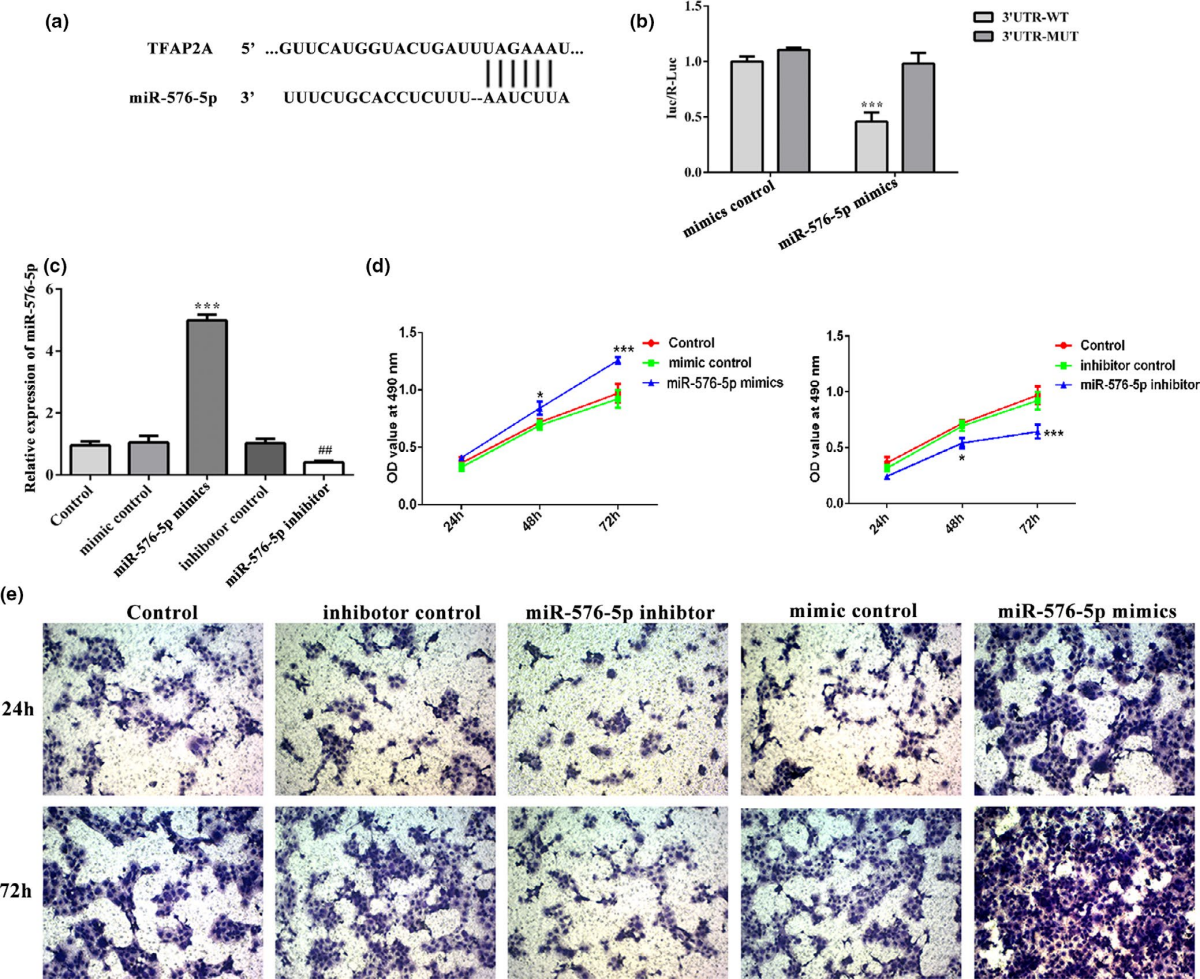
Luciferase reporter assay for target verification. (a) Gene target prediction obtained using bioinformatics software. (b) Luciferase activity in miR‐576‐5p mimic and mimic control groups. ***p < .01 versus 3'UTR‐WT. (c) RT‐qPCR assay was perfected to validate miR‐576‐5p mimics and inhibitor transfection efficiency. (d) OD at 490 nm was detected using cell counting kit‐8 (CCK‐8) assay at 24, 28, and 72 hr following transfection in control, mimic control, inhibitor control, and miR‐576‐5p mimic and miR‐576‐5p inhibitor groups. (e) Invasion activity in control, mimic control, inhibitor control, and miR‐576‐5p mimic and miR‐576‐5p inhibitor groups. Transwell assays were performed 24 and 72 hr posttransfection. Magnification, ×200. *p < .05 and ***p < .01 versus control and mimic control groups; ##*p* < .01 versus control and inhibitor groups. OD, optical density

The animal experiments in vivo have shown that miR‐576‐5p and TFAP2A are associated with the onset of PE, which may affect the invasion of trophoblast cells. However, the relationship between them is unclear. The complimentary sequences for miR‐576‐5p in the 3'UTR of TFAP2A were obtained from the TargetScan database (Figure 2a). After HTR8/SVneo cells were transfected with miR‐576‐5p mimic and TFAP2A 3'UTR‐WT, the luciferase activity was decreased sharply than that in cells transfected with 3'UTR‐MUT (*p* < .001; Figure 2b). These results indicated that TFAP2A is target protein of miR‐576‐5p.

### 3.4 Effects of miR‐576‐5p on cell viability and invasion of HTR8/SVneo cells

First, the transfection efficiency of miR‐576‐5p mimic in HTR8/SVneo cells was assessed using RT‐qPCR, miR‐576‐5p expression showed significantly elevated in miR‐576‐5p mimic group (*p* < .001) and decreased in miR‐576‐5p inhibitor group (*p* < .01) when compared with control, mimic control, and inhibitor control groups (Figure 2b). Then, results from CCK‐8 assay indicated that miR‐576‐5p mimics significantly promoted cell viability of HTR8/SVneo cells but miR‐576‐5p inhibitor reduced cell viability at 48 and 72 hr when compared with their plasmids control (*p* < .05 and *p* < .001; Figure 2d). Additionally, results from the Transwell assay showed that the number of invading cells was significantly reduced in HTR8/SVneo cells transfected with miR‐576‐5p inhibitor, and increased sharply in HTR8/SVneo cells transfected with miR‐576‐5p mimics, when compared with inhibitor or mimics control groups (Figure 2e).

### 3.5 Effects of TFAP2A on cell viability and invasion of HTR8/SVneo cells

**Figure 3:**
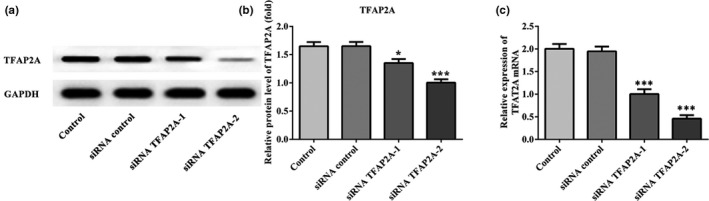
The transfection efficiency of siRNA TFAP2A‐1 and siRNA TFAP2A‐2 was assessed using western blot and RT‐qPCR assays. (a) The protein level of TFAP2A and (b) the mRNA level of TFAP2A in control, siRNA control, siRNA TFAP2A‐1, and siRNA TFAP2A‐2 groups. *p < .05 and ***p < .001 versus control

As seen in Figure 3, western blotting and RT‐qPCR were performed to verify the transfection efficiency of siRNA‐TFAP2A‐1 and siRNA‐TFAP2A‐2. The protein and mRNA level of TFAP2A markedly decreased in both the siRNA‐TFAP2A‐1 and siRNA‐TFAP2A‐2 groups (*p* < .05 and *p* < .001; Figure 3a and b). The siRNA‐TFAP2A‐2 plasmid has the best interference efficiency, which is chosen for subsequent experiments.

**Figure 4:**
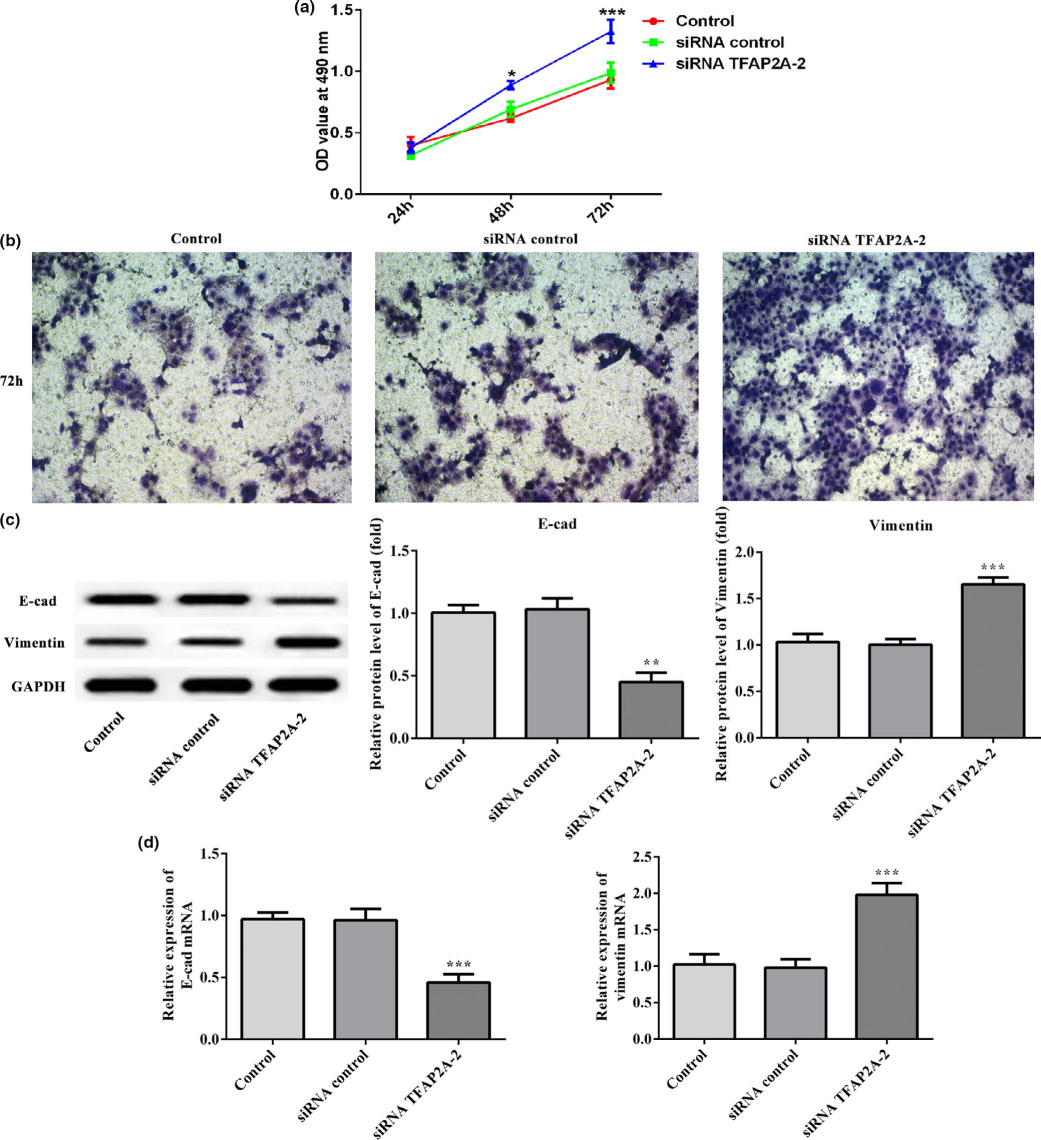
Effects of siRNA TFAP2A‐2 on cell activity and invasion, and the expression of E‐cad and Vimentin in HTR8/SVneo cells. (a) A CCK‐8 assay was used to detect cell activity among control, siRNA control, and siRNA TFAP2A‐2 groups. (b) Representative pictures of Transwell assay for invasion ability test. (c) Western blotting and (d) RT‐qPCR analysis were performed to detect the protein and mRNA levels of E‐cad and Vimentin among control, siRNA control, and siRNA TFAP2A‐2 groups. **p < .01 and ***p < .001

Next, the cell viability and invasion of HTR8/SVneo cells transfected with siRNA‐TFAP2A‐2 were significantly elevated when compared with control group (*p* < .05 and *p* < .001; Figure 4a and b). In addition, downregulation of TFAP2A reduced E‐cad expression and improved Vimentin in the protein and mRNA levels comparing to control group (*p* < .001; Figure 4c and d). These evidences suggest that TFAP2A has exactly the opposite effect on cell viability and invasion ability of HTR8/SVneo cells as miR‐576‐5p, and may play a certain role in the development of PE.

## 4 DISCUSSION

Preeclampsia is a major cause of maternal and fetal morbidity and mortality, which pathogenesis has always been the focus of research in recent years. Abnormal miRNA expression may be a cause of pregnancy complications including PE (Biro, Nagy, & Rigo, 2017). Increasing evidence suggests that miRNA may participate in the pathogenesis of PE via negatively regulating gene expression (Pineles et al., 2007; Zhu, Han, Sargent, Yin, & Yao, 2009). The most consistent evidence is the overexpression of miR‐210 in PE placentae, and its role is well established (Pineles et al., 2007). In the present study, we selected miR‐576‐5p as an RNA of interest based on RT‐qPCR results. The expression of TFAP2A was detected in placenta tissue from PE rats and the normal control placentas using western blotting and immunofluorescence assay. Previously, overexpression of miR‐576‐5p was observed in brain‐metastatic carcinomas (Li, Gu, Fang, Xiang, & Chen, 2012); however, miR‐576‐5p was downregulated in osteoarthritis (OA) chondrocyte pellets (Diaz‐Prado et al., 2012). Meanwhile, miR‐576‐5p regulate the migration and invasion of esophageal squamous cell carcinoma cells by inhibiting NRIP1 expression (Ni et al., 2018). Subsequently, a dual‐luciferase reporter assay showed that TFAP2A was a direct target of miR‐576‐5p and its expression was negatively correlated with the expression of miR‐576‐5p.

Defective trophoblast invasion is the leading cause of PE. One study showed that miR‐200a and miR‐141 weaken the ERK signaling, thereby suppressing the migration and invasion of trophoblast cells by targeting EG‐VEGF in PE (Wang et al., 2019). Another research indicated that miR‐181a‐5p targeted the insulin‐like growth factor 2 mRNA‐binding protein 2 (IGF2BP2), which contain a highly conserved miR‐181a‐5p‐binding site within the 3′UTR, thereby affecting the invasion and migration of cytotrophoblasts (Wu et al., 2018). A study revealed novel functions of miR‐576‐5p in regulating cell migration and invasion in esophageal squamous cell carcinoma (ESCC) (Ni et al., 2018). Consistently, the expression of miR‐576‐5p was downregulated in placentas of PE rats compared with normal control placentas, as well as TFAP2A was identified as the miR‐576‐5p target in our study. The increased cell invasiveness in the placental tissue was again demonstrated with E‐cad overexpression and Vimentin downregulation in the present study (Figure 1). Here, silence of miR‐576‐5p markedly inhibited cell activity and invasion of HTR8/SVneo cells; however, overexpression of miR‐576‐5p increased cell activity and invasion (Figure 2). These results indicated that miR‐576‐5p is an inhibitory factor involving in the pathogenesis of PE.

Earlier studies showed that the transcription factor AP‐2α (activator protein‐2 alpha, also called TFAP2A) played an important role in terminal differentiation of cytotrophoblast cells (Cheng et al., 2004). Kotani et al. (2009) found that overexpression of TFAP2A inhibited the migration and invasion of HTR8/SVneo cells. In addition, TFAP2A‐mediated regulation of Bcl‐2 and Bax regulation influences apoptosis which in turn lead to the pathogenesis of PE (Zhang et al., 2012). TFAP2A suppressed trophoblast invasion by repression of MMP‐2 and MMP‐9 and upregulation of E‐cadherin, thus leading to shallow placentation in severe PE (Zhang et al., 2013). The present results demonstrated that knockdown of TFAP2A in HTR8/SVneo cells caused a dramatic increase in the cell proliferation and invasion abilities. Meanwhile, the expression of E‐cad was decreased and Vimentin was elevated significantly following siRNA TFAP2A treatment. Therefore, TFAP2A, may be a pathogenic gene, increases the severity of PE by inhibiting cell invasion in placenta tissue. Interfering with TFAP2A expression may be an effective treatment for PE.

There are some limitations of the present study. First, the pathoetiology of PE is complex, it cannot exclude that other factors may be involved in the influence of miR‐576‐5p/TFAP2A axis in PE. Moreover, an appropriate animal model mimicking PE may be able to elucidate the causative role of miR‐576‐5p/TFAP2A in the disease.

## 5 CONCLUSION

We found that abnormal expression of miR‐576‐5p and TFAP2A in rat models of PE and miR‐576‐5p affected the process of PE via regulating TFAP2A expression. Our findings indicated that miR‐576‐5p suppressed cell proliferation and invasion of human trophoblast cells in vitro by negatively regulating the TFAP2A expression and could be further investigated as disease biomarkers and therapeutic targets in future studies.

## ETHICS APPROVAL AND CONSENT TO PARTICIPATE

All animal experiments have been approved by the Local Ethics Committee of the 987 hospital of the PLA joint logistics support force. All animals received humane care and the experimental procedures were conducted in strict accordance with the health and care guidelines for experimental animals.

## CONFLICT OF INTEREST

All the authors declared no competing interests.
